# Cutaneous wound healing in type 2 diabetes *db/db* mice was impaired with specific changes in proinflammatory cytokine expression

**DOI:** 10.1007/s00403-025-03883-y

**Published:** 2025-02-08

**Authors:** Kanae Mukai, Arya Iswara, Toshio Nakatani

**Affiliations:** 1 Faculty of Health Sciences, Institute of Medical, Pharmaceutical and Health Sciences, Kanazawa University, 5-11-80 Kodatsuno, Kanazawa, 920-0942 Japan; 2 Division of Health Sciences, Graduate School of Medical Sciences, Kanazawa University, Kanazawa, Japan; 3 Faculty of Nursing and Health Sciences, Universitas Muhammadiyah Semarang, Semarang, Indonesia

**Keywords:** Cutaneous wound healing, Diabetes mellitus, Nonhealing, Cytokines

## Abstract

In *db/db* mice, cutaneous wound healing was delayed, and excessive wound exudates enlarged the wound. However, the relationship between enlarged wounds and proinflammatory cytokine expression remains unknown. Therefore, we investigated the expression of proinflammatory cytokines *Tnf-α* and *Il-6* in cutaneous wound healing with diabetes. In this study, 12 C57BL/6J mice (wild-type: WT) and 14 *db/db* mice were subjected to full-thickness wound injuries. Wound healing was assessed until day 14, and wound tissues were harvested on days 7, 9, 11, and 14. The wound areas increased for 4 days, gradually increased until day 9, and stabilized until day 14 in the *db/db* group, but increased for 3 days, rapidly decreased until day 12, and gradually decreased until day 14 in the WT group. On day 14, the wound area in the *db/db* group was significantly larger than that in the WT group (*p* < 0.01). The relative expressions of the *Tnf-α* and *Il-6* in the *db/db* group were significantly higher than those in the WT group on days 7–14, and on days 11 and 14, respectively (*p* < 0.05). Our study showed that cutaneous wound healing was delayed with wound expansion and the expression of *Tnf-α* and *Il-6* was high throughout the measurement time points in *db/db* mice. These abnormal expressions could influence impaired cutaneous wound healing in diabetic mellitus.

**Supplementary Information:**

The online version contains supplementary material available at 10.1007/s00403-025-03883-y.

## Introduction

Diabetes mellitus (DM) has emerged as one of the most serious and common chronic diseases, causing life-threatening, disabling and costly complications, and reducing life expectancy [1]. A global diabetes prevalence is expected to reach 783.2 million individuals (12.2%) aged 20–79 years old by 2045 [2]. Diabetic foot is a common complication associated with DM that presents as deep lesions of tissues intermingled with neurological disorders and peripheral vascular disease of the lower limbs. A recent systematic review and meta-analysis reported that 6.3% (95% Cl: 5.4–7.3%) of individuals with DM worldwide will develop diabetic foot ulcer (DFU) over the course of their lifetime [3]. Approximately 20% of individuals who develop DFU will require lower-extremity amputation [4], and 10% will die within 1 year from the initial diagnosis of DFU [5, 6]. Furthermore, DFU significantly influences the patient’s quality of life [7, 8] and economic burden [9]. Impaired wound healing is a hallmark of the pathophysiology of DFUs and limb amputations. Despite the magnitude of these clinical consequences, the mechanism by which DM impairs cutaneous wound healing remains unknown.

Cutaneous wound healing is a complex and tightly orchestrated response to injury, is carefully regulated at the temporal and spatial levels, and involves the following three major overlapping phases: inflammation, proliferation, and remodeling [10], which allows for the repair of damaged or injured tissues. Meanwhile, DFUs lead to delayed cutaneous wound healing [11]. Inflammation and tissue response to wounds are abnormal in experimentally diabetic wounds [12–14]. Diabetic wounds are characterized by excessive and prolonged inflammation by increasing the release of proinflammatory cytokines, such as tumor necrosis factor (TNF)-α and interleukin (IL)-1β from peritoneal macrophages (12 h after lipopolysaccharide or IFN-γ application) [15], TNF-α and IL-6 in the wound tissue (days 1, 3, and 7 after wounding) [16], and TNF-α in wound tissue (day 4 after wounding) [17]. However, these previous studies only focused on the inflammatory phase or limited time points, so the exact reactions causing this excessive and prolonged inflammation throughout the whole cutaneous wound healing process are poorly understood.

Our previous studies reported delayed cutaneous wound healing in the *db/db* mice due to expansion of the wound area throughout the whole cutaneous wound healing process [18, 19]. Moreover, our recent study showed that excessive wound exudates in the *db/db* mice lead to wound enlargement throughout the observation [20]. Wound exudates keep the wound moist, supply nutrition, and provide the medium for epithelial cell migration and mitosis. Meanwhile, excessive wound exudates can cause maceration of the surrounding skin. Maceration may increase the risk of friction damage and skin breakdown [21]. These findings lead to a potentially novel insight that excessive wound exudates in the *db/db* mice caused maceration of the surrounding skin, thereby leading to wound enlargement, followed by an inflammatory response, and delayed cutaneous wound healing. One aspect of diabetic wound healing receiving considerable attention is the enhanced and prolonged expression of potent proinflammatory cytokines, such as TNF-α and IL-6 [22]. However, the relationship between these enlarged wounds throughout the cutaneous wound healing in the *db/db* mice and proinflammatory cytokine expression remains unknown. Therefore, we investigated the expression of proinflammatory cytokines *Tnf-α* and *Il-6* in enlarged wounds on cutaneous wound healing in individuals with diabetes.

## Materials and methods

### Animals

We included 12 10-week-old female C57BL/6J mice and 14 10-week-old female *db/db* mice (C57BLKS/J Iar-+Lepr^db^/+Lepr^db^) with diabetes (Sankyo Lab Service Co., Tokyo, Japan). They were individually placed in an air-conditioned room at 25.0 °C ± 2.0 °C with lights turned on at 08:45–20:45, and water and chow were freely provided. The Kanazawa University Animal Experiment Committee reviewed and approved all animal experiments, which were conducted following the Guidelines for the Care and Use of Laboratory Animals of Kanazawa University, Japan (AP-204120).

### Wound and blood glucose assay

The mice were anesthetized using 1.5–2.0% isoflurane (Wako, Tokyo, Japan) at 1.5 L of O_2_/min via a plastic tube mask. The dorsum was shaved, and the mice were divided into wild-type (WT) and *db/db* groups. One day following dorsum shaving, two circular full-thickness skin wounds (4-mm diameter) in the panniculus carnosus muscle were created on both sides of the dorsum by using Kai sterile disposable biopsy punch (Kai Industries Co. Ltd., Gifu, Japan) under inhalational anesthesia. To maintain a moist environment, the wounds were covered with a hydrocolloid dressing (Tegaderm; 3 M Health Care, Tokyo, Japan), which was changed daily. An aliquot of blood was obtained from the tail vein, and blood glucose level (mg/dL) was monitored using an automatic glucometer (Carefast^®^, NIPRO, Tokyo, Japan). Blood glucose level and body weight were monitored daily until day 14 after wounding.

### Macroscopic observations

Day 0 was defined as the day when wounds were created, and the wound healing process was assessed until day 14 following wounding. The wound edges were traced using polypropylene sheets, and the images were obtained daily under inhalational anesthesia. We captured the traces on the sheets using a scanner on a personal computer using Adobe Photoshop Elements 24.3 (Adobe System Inc., Tokyo, Japan) and calculated the wound areas using ImageJ (National Institutes of Health, Bethesda, MD, USA). Based on our previous studies, the wound area was presented as the ratio of the daily wound area to the initial wound area on day 0 [18, 19, 23].

### Tissue and serum collection

The mice were euthanized by isoflurane overdose on days 7, 9, 11, and 14 after wounding. The wound and surrounding intact skin were harvested, and each wound and surrounding intact skin sample was bisected at the center. Before fixing for histology, one-half of each wound was embedded in tissue-Tek OCT (Sakura Finetek Japan Co. Ltd., Tokyo, Japan). The remaining half was snap-frozen in liquid nitrogen and stored at − 80 ℃ before RNA isolation. Serum was collected from blood isolated through cardiac puncture on day 14 after wounding and stored at − 20 ℃ until the time of assay.

### Histological staining and microscopic observations

Sections near the center of the wound were obtained from one wound and stained. Subsequently, 5–10-µm-thick ice sections near the center of the wound were subjected to hematoxylin and eosin (HE) staining. HE staining images were imported onto a computer using a digital microscopic camera (DP27-CU; Olympus, Tokyo, Japan). The re-epithelialization ratio (re-epithelialization length/wound length) was assessed using DP27-CU (Olympus, Tokyo, Japan) [20].

### RNA isolation and real-time polymerase chain reaction

RNA was isolated from the whole-wound homogenate using the Purelink RNA mini kit (ThermoFisher Scientific K.K., MA, USA). cDNA was transcribed using the PrimeScript RT reagent kit with gDNA eraser (TAKARA Bio Inc., Shiga, Japan). Quantitative real-time PCR was performed using TB Green Premix Ex Taq II (TAKARA Bio Inc., Shiga, Japan) and an AriaMx Real-Time PCR system (Agilent Technologies Inc., CA, USA). Each sample was serially diluted over three orders of magnitude, and expression levels were normalized to those of the *Gapdh* housekeeping gene. The primer sequences (*Tnf-α*, *Il-6*, and *Gapdh*) are listed on the supplement sheet (Table S1).

### Serum insulin assay

Serum insulin levels were determined using ELISA (10-1247-01; Mercodia AB, Uppsala, Sweden) following the manufacturer’s guidelines. The plate was read using a microplate reader MTP-310 Lab System (Corona Electric Co. Ltd., Ibaraki, Japan) at 450 nm.

### Statistical analysis

Data were expressed as means ± standard error of the mean (SEM) and analyzed using JMP^®^ 12.1.0 (SAS Institute Inc., Cary, NC, USA). The t-test was used to compare the two groups. A *p* value of *<* 0.05 was considered statistically significant.

## Results

### Body weight, blood glucose, and serum insulin levels

**Fig. 1:**
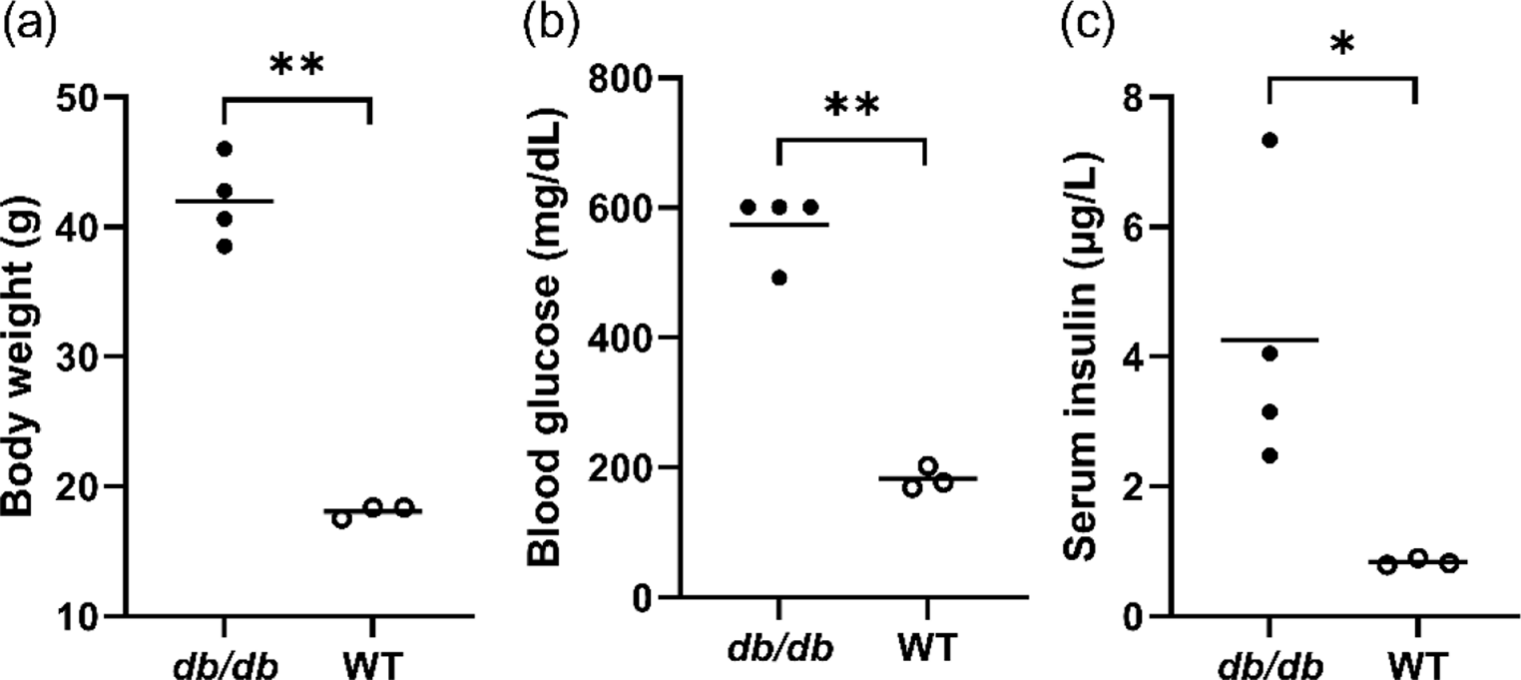
Body weight, blood glucose, and serum insulin in the experimental mice. (**a**) Body weights are shown as a dotted graph on day 14 after wounding. (**b**) Blood glucose levels are shown as a dotted graph on day 13 after wounding. (**c**) Serum insulin levels are shown as a dotted graph on day 14 after wounding. Values are expressed as means ± SEM, *n* = 3–4 mice: Student’s t-test, **p < 0.01 and * *p* < 0.05

The body weight was 42.0 ± 2.3 g in the *db/db* group and 18.1 ± 0.3 g in the WT group on day 14 after wounding, and it was significantly higher in the *db/db* group than that in the WT group (*p* < 0.01) (Fig. 1a). Blood glucose level was 573.8 ± 38.5 and 182.7 ± 9.9 mg/dL in *the db/db* and WT groups, respectively, on day 13 after wounding, and it was significantly higher in the *db/db* group than in the WT group (*p* < 0.01) (Fig. 1b). The serum insulin level was 4.25 ± 1.08 and 0.83 ± 0.03 µg/L in *the db/db* and WT group, respectively, on day 14 after wounding, and it was significantly higher in the *db/db* group than in the WT group (*p* < 0.05) (Fig. 1c).

### Macroscopic observations and wound areas

**Fig. 2:**
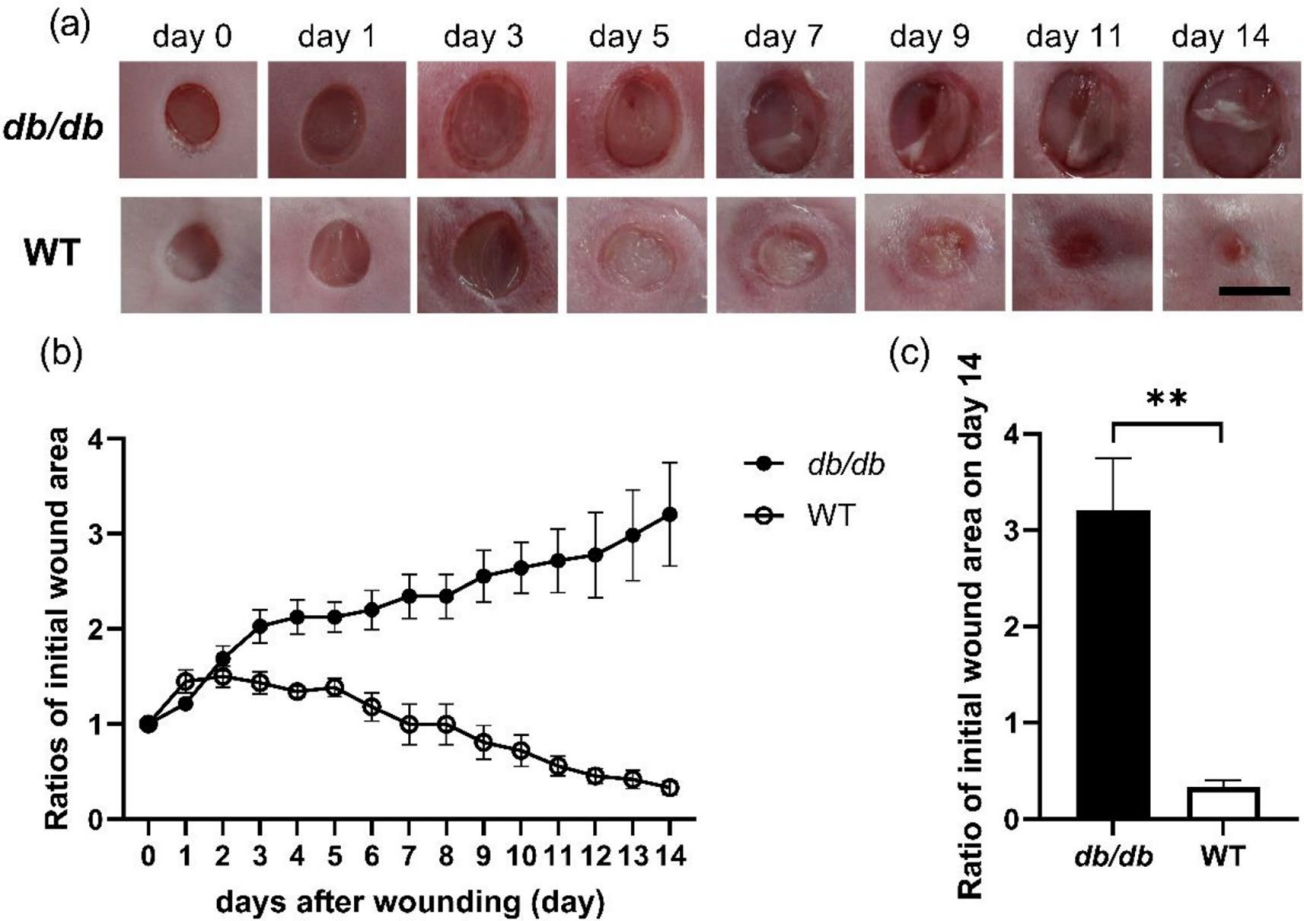
Macroscopic wound healing. (**a**) Wounds with a 4-mm diameter are created, and images are obtained to assess wound healing. Bar, 5 mm. The wound area-to-the-initial area ratios on day 0 are shown as line graphs based on each day (**b**) and as a box graph on day 14 after wounding (**c**). Values are expressed as means ± SEM, *n* = 6–8 wounds: Student’s t-test, **p < 0.01

The wound areas in the *db/db* group increased for 4 days (on day 4, the ratio of the wound area to the initial wound area was 1.92 ± 0.15). They gradually increased until day 9 (2.41 ± 0.30) and stabilized until day 14 (2.76 ± 0.54). The wound surface was not covered with a new epithelium until day 14. Conversely, the wound areas in the WT group increased for 3 days (1.43 ± 0.12) and rapidly decreased until day 12 (0.46 ± 0.08). Subsequently, they gradually decreased until day 14 (0.33 ± 0.07), and the wound surface was covered with a new epithelium (Fig. 2a and b, and supplemental Fig. S1). On day 14 after wounding, the wound area in the *db/db* group was significantly larger than that in the WT group (*p* < 0.01) (Fig. 2c).

### Re-epithelialization

**Fig. 3:**
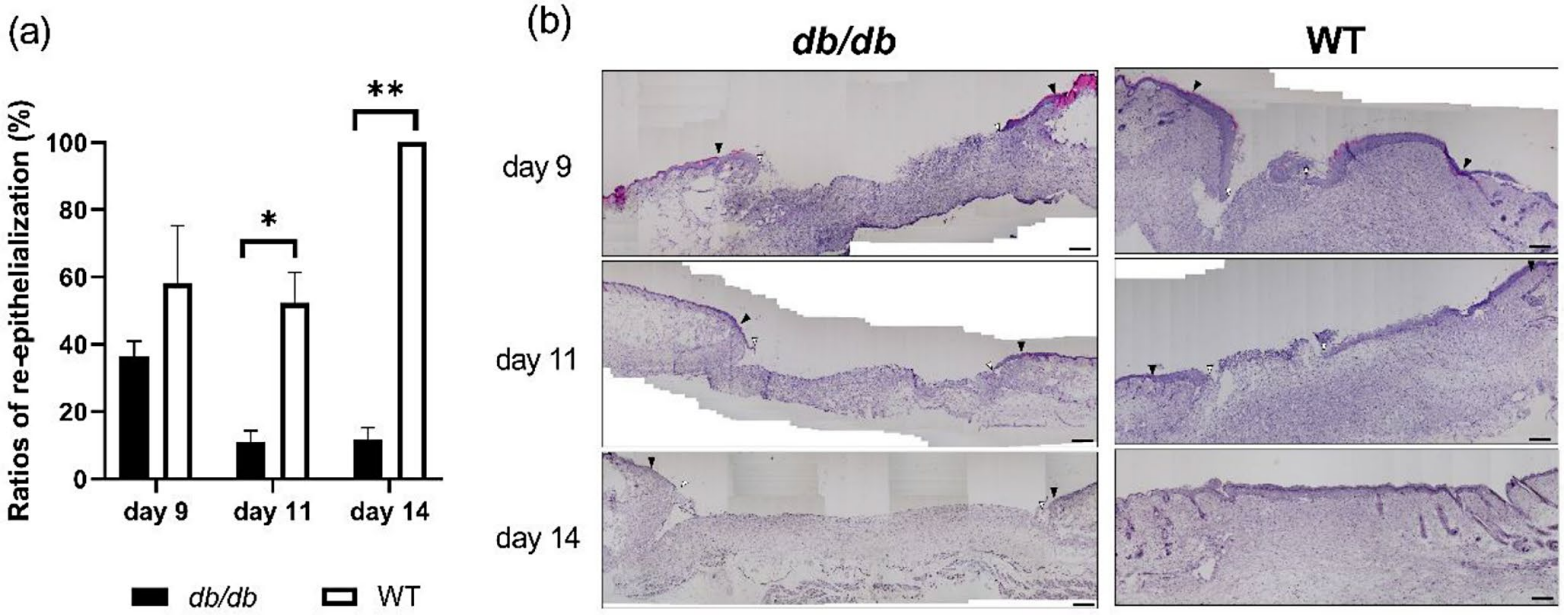
Re-epithelialization. (**a**) The ratio of re-epithelialization (%) on days 9, 7, and 14 after wounding is depicted as a box graph. Values are expressed as means ± SEM, *n* = 6–8 wounds: Student’s t-test, **p < 0.01 and * *p* < 0.05. (**b**) Hematoxylin and eosin staining (bars, 200 μm) of granulation tissues on days 9, 11, and 14. Arrows with black indicate wound edges, and arrows with white indicate re-epithelium edges

In the WT group, the new epithelium gradually grew from the wound edges, and they almost completely covered the wound surface until day 14. In the *db/db* group, it gradually grew from the wound edges temporarily on day 9. However, the new epithelium did not elongate as the wound re-expanded, and they did not cover the wound surface until day 14. The ratios of re-epithelialization in the *db/db* group were significantly lower than those in the WT group on days 11 and 14 (*p* < 0.05 and *p* < 0.01, respectively) (Fig. 3a and b).

### Relative expression of inflammatory cytokines, such as *Tnf-α* and *Il-6*

**Fig. 4:**
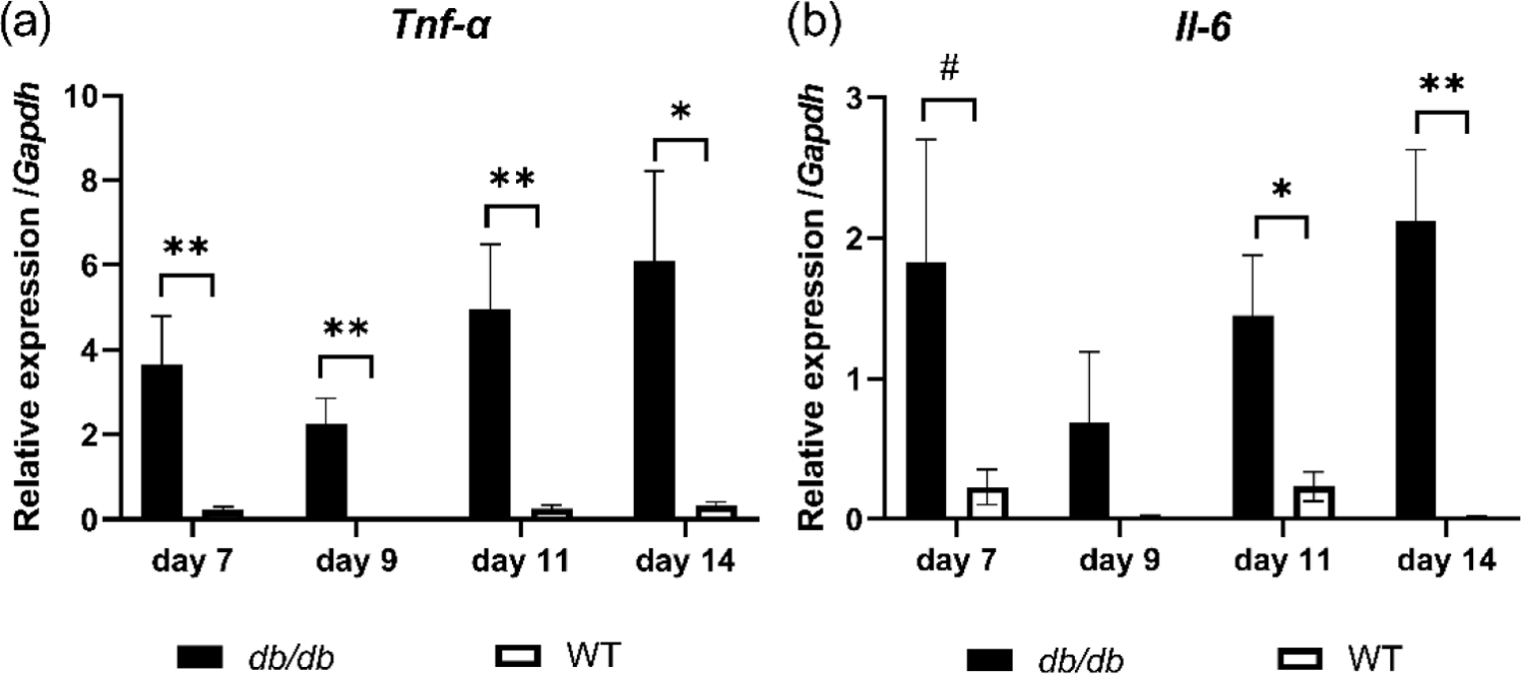
Relative expression of the proinflammatory cytokines. (**a**) The relative expression of *Tnf-α* is shown as a box graph on days 7, 9, 11, and 14 after wounding. (**b**) The relative expression of *Il-6* is shown as a box graph on days 7, 9, 11, and 14 after wounding. Values are expressed as means ± SEM, *n* = 5-8 wounds: Student’s t-test, ^#^*p* < 0.1, **p < 0.01, and * *p* < 0.05

In the WT group, the relative expressions of the inflammatory cytokines, such as *Tnf-α* and *Il-6*, were low throughout the measurement time points. Conversely, in the *db/db* group, these were high throughout the measurement time points and showed specific changes associated with wound expansion, but were temporarily low until day 9 and high on days 11 and 14. The relative expression of the *Tnf-α* in the *db/db* group was significantly higher than that in the WT group on days 7, 9, 11 (*p* < 0.05), and 14 (*p* < 0.01) (Fig. 4a). The relative expression of *Il-6* in the *db/db* group was significantly higher than that in the WT group on days 11 (*p* < 0.05) and 14 (*p* < 0.01) and tended to be higher than that in the WT group on day 7 (*p* < 0.1) (Fig. 4b).

## Discussion

In the *db/db* mice, cutaneous wound healing was delayed by expanding the wound area throughout the entire cutaneous wound healing process [18, 19], and excessive wound exudates in the *db/db* mice led to wound enlargement throughout the observation [20]. These findings lead to a potentially novel insight that excessive wound exudates in the *db/db* mice caused maceration of the surrounding skin, thereby leading to wound enlargement, an inflammatory response, and delayed cutaneous wound healing. Moreover, diabetic wound healing involves enhanced and prolonged expression of potent proinflammatory cytokines, such as TNF-α and IL-6 [22]. However, the relationship between enlarged wounds throughout the whole cutaneous wound healing process in the *db/db* mice and proinflammatory cytokine expression remains unknown. Therefore, we investigated the expression of proinflammatory cytokines *Tnf-α* and *Il-6* in enlarged wounds on cutaneous wound healing in diabetes. Consequently, our study showed that cutaneous wound healing was delayed with wound expansion and the expression of proinflammatory cytokines *Tnf-α* and *Il-6* was high throughout the measurement time points in *db/db* mice.

DM is a disorder wherein the body does not produce enough or respond normally to insulin, causing blood glucose levels to be abnormally high. Various animal models have been established: chemically induced, spontaneous autoimmune, genetically induced, or high-fat feeding [24, 25]. Genetically induced *db/db* or *ob/ob* mice are widely used in the field of cutaneous wound healing in patients with diabetes. Moreover, comparative research reported that *db/db* mice exhibit impaired wound healing compared with other murine strains with diabetes [26]. Hence, the current study selected *db/db* mice as a diabetic model. Previous studies have shown that mice with a blood glucose level of > 250 mg/dL were considered to have DM [27], and the blood glucose level in *db/db* mice was > 400 mg/dL after 8 weeks [18, 19, 28]. This study showed that the blood glucose levels were 182.7 ± 9.9 and 573.8 ± 38.5 mg/dL in the WT and *db/db* groups, respectively. Additionally, the serum insulin levels in the *db/db* group were significantly higher than those in the WT group. The serum insulin levels in *db/db* mice were high compared with nondiabetic mice [28]. Our animal model had valid diabetes with hyperglycemia and insulin resistance.

Delayed chronic wounds, such as DFU, remained in the early inflammatory phase within the cutaneous wound healing process, which did not favor tissue regeneration [29], and the proliferative and remodeling phases did not readily occur [30]. Therefore, targeting prolonged inflammation in DFU may be an effective method to revert to the normal cutaneous wound healing process. The main key finding of this study is the high *Tnf-α* and *Il-6* expression throughout the measurement time points in diabetic wounds. Impaired cutaneous wound healing in patients with DM is accompanied by decreased early inflammatory cell infiltration but increased numbers of neutrophils and macrophages in the late phases. These changes in inflammatory cell recruitment occurred in conjunction with alterations in chemokine and growth factor expression [31]. In the diabetic models, increased TNF-α and IL-6 levels were observed in diabetic wound tissue compared with the nondiabetic healed wound tissue [16]. TNF-α is a non-interleukin cytokine that plays a role in cutaneous wound healing and is synthesized mainly by macrophages. Previous studies reported increased TNF-α level in aged human subjects, and TNF-α level and *Tnf-a* expression in murine model of delayed cutaneous wound healing [32]. Additionally, *Tnf-α* expression was higher in diabetic mice wounds than normal mice wounds [17]. Moreover, high glucose levels stimulated macrophages to enhance *Tnf-α* expression [33]. This study showed that the wound areas in the *db/db* group increased until day 9 and stabilized until day 14, which was the endpoint, and the relative *Tnf-α* expressions in the *db/db* group were significantly higher than those of the WT group on days 7, 9, 11, and 14. In normal wound repair, TNF-α level began to elevate rapidly after wounding and reached a peak [34]. Therefore, abnormal expression in diabetic wounds, which have temporary high *Tnf-α* expression in the *db/db* group highly impacted the cutaneous wound healing process, and this abnormal expression could influence impaired cutaneous wound healing. Moreover, IL-6 is an interleukin cytokine secreted by T cells and macrophages. A previous study showed that IL-6 controlled cutaneous wound healing. Delayed cutaneous wound healing in *Il-6*-deficient transgenic mice was reversed with a single dose of recombinant murine IL-6 or intradermal injection of an expression plasmid containing the full-length murine *Il-6* cDNA [35]. This study showed that the wound areas in the *db/db* group increased until day 9 and stabilized until day 14, which was the endpoint, and the relative expressions of *Il-6* in the *db/db* group were significantly higher than those of the WT group on days 11 and 14. In normal wound repair, IL-6 level reached a peak late in TNF-α level [34]. Therefore, the temporarily elevated *Il-6* expression in the *db/db* group, could influence impaired cutaneous wound healing.

This study has four limitations. First, the experiments were only conducted using *db/db* mice, a type-2 DM animal model. Whether similar results could be obtained in other models, such as the type 1 DM animal model remains unclear. Second, the wounds in this experiment were covered with HCD dressing. Whether similar results could be obtained from other materials remains unclear. Third, the wounds in this experiment were monitored until day 14 after the wounding. Whether the ratio of the wound area to the initial wound area in *db/db* mice consistently stable over several days after day 14 remains unclear. Finally, the expression analysis performed only RNA levels. It’s unclear if protein expression could produce comparable outcomes. More research is required to resolve these viewpoints in the near future.

## Conclusion

Our study showed that cutaneous wound healing was delayed with wound expansion and the expression of proinflammatory cytokines *Tnf-α* and *Il-6* was high throughout the measurement time points in *db/db* mice. These abnormal expressions could influence the impaired cutaneous wound healing in diabetic mellitus.

## Electronic supplementary material

Below is the link to the electronic supplementary material.

Supplementary Material 1

## Data Availability

No datasets were generated or analysed during the current study.
