# Supplementary material for: Cutaneous wound healing in type 2 diabetes db/db mice was impaired with specific changes in proinflammatory cytokine expression
Source: Arch Dermatol Res. 2025 Feb 8;317(1):367. doi: 10.1007/s00403-025-03883-y (PMC11807023; doi:10.1007/s00403-025-03883-y)
Supplement: Supplementary file 1 — Supplementary Material 1 [file 403_2025_3883_MOESM1_ESM.pdf]

Supporting information

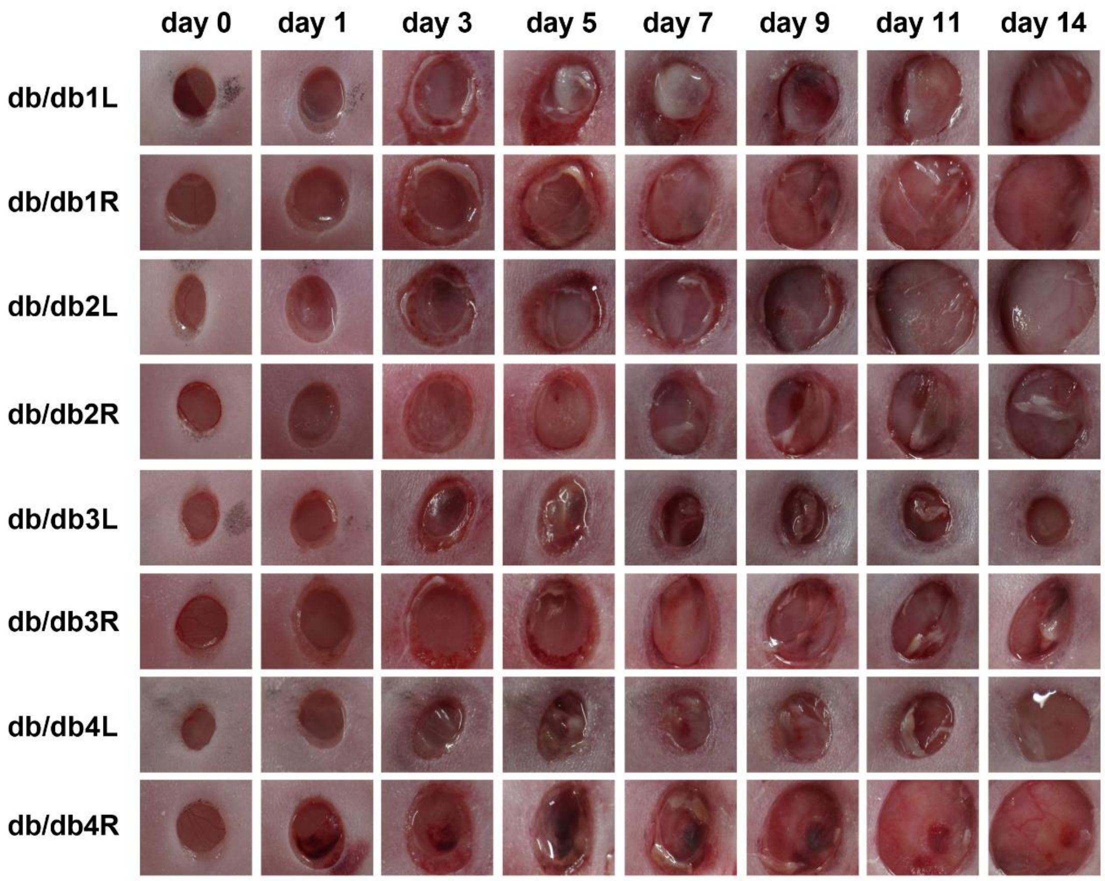

Fig S1. Macroscopic wound healing in *db/db* mice in this experiment.

Wounds with 4-mm diameter are created, and images are obtained to assess wound healing.

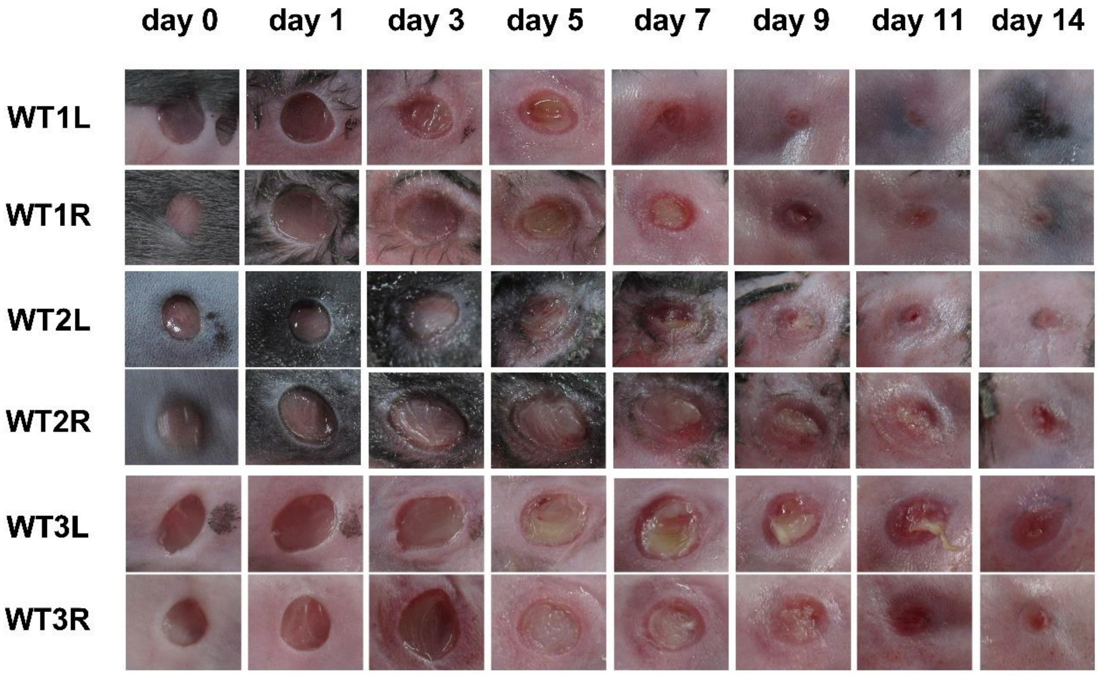

Fig S2. Macroscopic wound healing in WT mice in this experiment.

Wounds with 4-mm diameter are created, and images are obtained to assess wound healing.

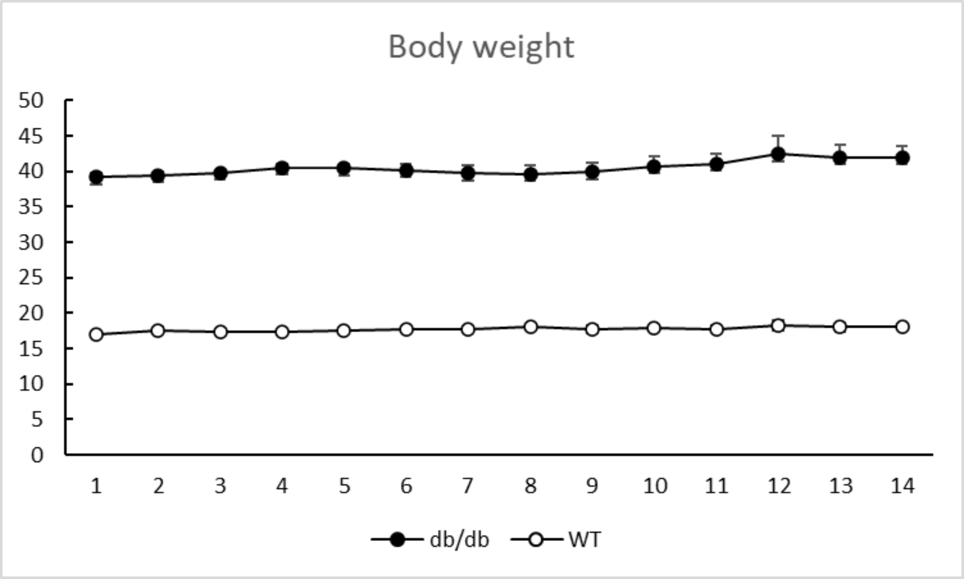

Fig S3. Body weights (g) among wound observation in *db/db* and WT groups.

N = 3-4 mice per group.

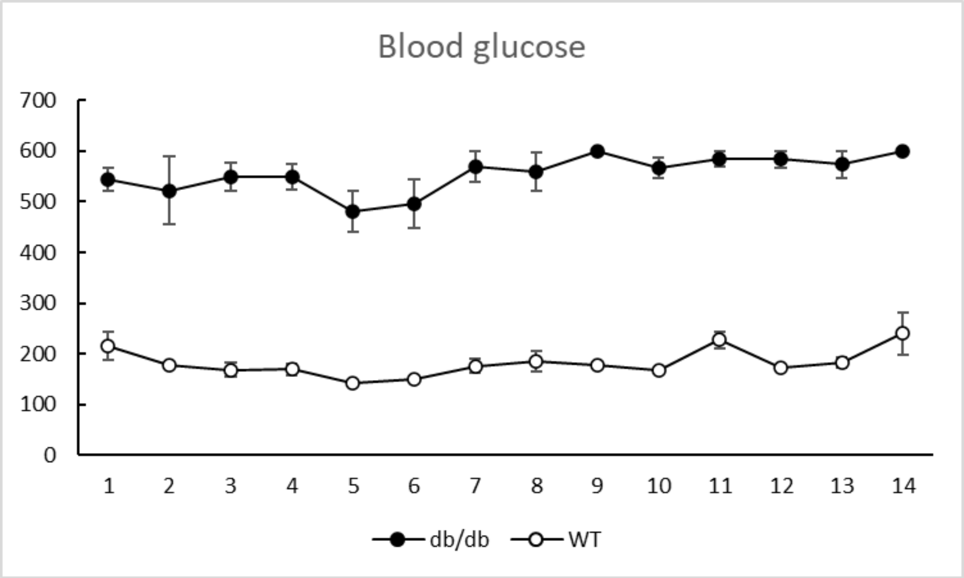

Fig S4. Blood glucose levels (mg/dL) among wound observation in *db/db* and WT groups.

N = 3-4 mice per group.

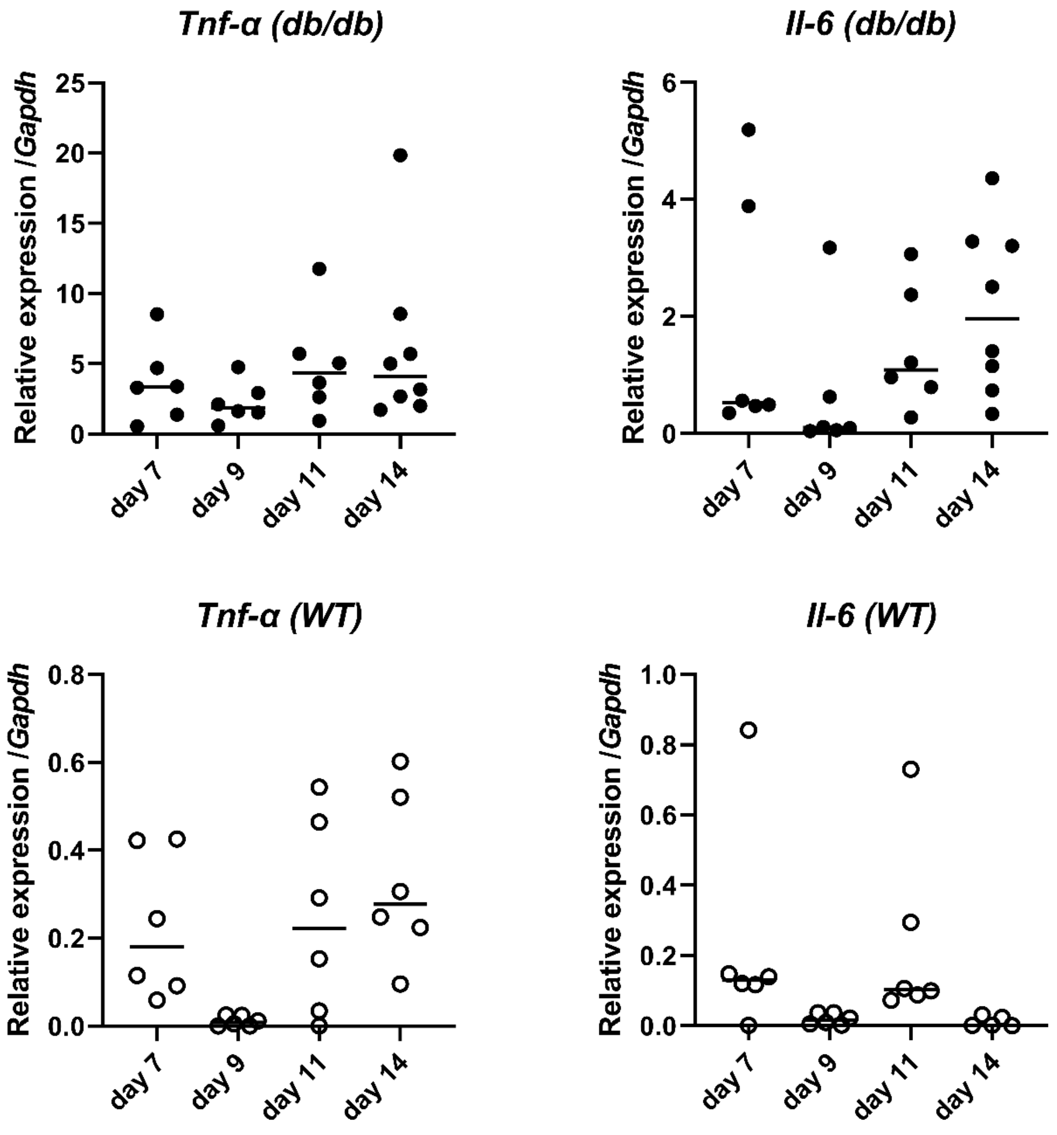

Fig S5. Relative expression of the proinflammatory cytokines within groups.

N = 5-8 mice per group.

Table S1. Primers in this experiment.

Name	Target	Forward primer	Reverse Primer
Tumor necrosis factor-α (*Tnf-α*)	Mouse	5'-ACGTCGTAGCAAACCACCAA-3'	5'-AAGGTACAACCCATCGGCTG-3'
Interleukin-6 (*Il-6*)	Mouse	5'-CCGGAGAGGAGACTTCACAG-3'	5'-TCCACGATTTCCCAGAGAAC-3'
Gapdh	Mouse	5'-TGATGGGTGTGAACCACGAG-3'	5'-GGCATGGACTGTGGTCATGA-3'
